# Effects of a group mindfulness-based cognitive programme on smartphone addictive symptoms and resilience among adolescents: study protocol of a cluster-randomized controlled trial

**DOI:** 10.1186/s12912-021-00611-5

**Published:** 2021-06-05

**Authors:** Anson Chui Yan Tang, Regina Lai Tong Lee

**Affiliations:** 1 School of Nursing, Tung Wah College, Hong Kong, China; 2 School of Nursing and Midwifery, Faculty of Health and Medicine, The University of Newcastle, 2308 Callaghan, New South Wales Australia

**Keywords:** smartphone addiction, smartphone behavior, adolescents, mindfulness, cognitive therapy, cluster randomized controlled trial, nursing

## Abstract

**Background:**

Smartphone addiction in adolescent is a significant global health issue since the last decade. Evidence has shown that the uncontrolled use of smartphone would lead to undesirable impact on their growth and development. However, evidence-based interventions to manage adolescents’ smartphone addictive behaviors is lacking. The proposed study aims to examine the effect of a group mindfulness-based cognitive programme(MBCP) on resilience, smartphone behavior and addictive symptoms in young adolescents.

**Methods:**

It is an open-label, parallel-group, cluster-randomized controlled trial with repeated measurement analysis. Four primary schools in Hong Kong will be recruited and randomly allocated in a ratio of 1:1 to the intervention/control group. A convenience sample of 240 class level 5 primary school students, 60 from each school, will be recruited. Participants in the intervention group will receive a 12-week MBCP which comprises 90-minute supervised practice at school and daily home practice. Resilience will be measured by Connor-Davidson Resilience Scale – 25 (Chinese version); smartphone behavior will be represented by time spent/day using the smartphone and types of functions used; addictive symptoms will be measured by Smartphone Addiction Scale-Short Version (Chinese Version). Baseline assessment(T0) will be conducted before the intervention starts. Post-tests will be conducted in weeks 4, 8, 12 of the intervention, and 3 months follow-up. Intention-to-Treat analysis will be applied to the variables. Generalized Estimating Equation model will be used to compare differences in resilience scores, smartphone behavior and addiction scores between and within groups, adjusted for socio-demographic factors. *P* < 0.05 with two-tailed test will be regarded as significance.

**Discussion:**

It is expected that adolescents will demonstrate better resilience and lesser smartphone addictive symptoms after joining the MBCP. The study will be the first provided empirical evidence to support the promising application of MBCP to manage smartphone use among adolescents. It introduces community stakeholders including community nurses a non-invasive and simple-to-administer intervention to tackle problematic smartphone use among adolescent clients.

**Trial registration:**

Chinese Clinical Trial Registry, ChiCTR2000033273, Registered on 26 May 2020.

## Background

The smartphone has become indispensable in the lives of most of humankind during the last two decades [1, 2]. More than 75 % of families own mobile devices such as smartphones [3]. Recent statistics from local and national surveys reveal a rapidly increasing trend in the use and possession of smartphones not only among adults but also among children and adolescents [4]. In 2011, 52 % of children below 9 years of age had access to mobile devices; in 2013, that access rate had increased to 75 % [5]. In 2018, about half of the elementary school students in the United State and Europe, possessed their own mobile phone [6]. In 2009, the average screen time of 8- to 18-year-old youngsters was 7.38 h, which is 1.17 h more than the one reported in 1999 [7]. A similar pattern has been observed in Hong Kong. A survey about the use of smartphones among 10- to 24-year-old adolescents and young adults revealed that 88.8 % of the respondents possessed a smartphone [8]. The older the age, the higher the possession rate [1]. The average time spent on screen media increased 15 % in nine years’ time, from 2003 to 2012. In particular, young adolescents aged 10 to 14 years showed the largest increase in screen time. Their average duration spent on screen media per week increased from 14.6 h to 2003 to 18.5 h in 2013 (an increase of nearly 27 %) [4].

The continuously increasing possession and use of mobile devices among children and adolescents is alarming because both their bodies and minds have not yet fully developed; in particular, their self-discipline is weak. The uncontrolled use of these mobile devices will make them even more susceptible than adults to the negative effects of mobile devices on their physio-psycho-social health [1]. Preliminary evidence shows that prolonged engagement with these devices disrupts cognitive development and leads to various physiological and psychosocial problems, including but not limited to visual problems, disruptions of memory, decline of attention, addiction, learning and social problems [1, 9, 10]. Furthermore, breaking addictive patterns early in life will help them maintain the benefits into adulthood. The research team therefore focuses on investigating effective intervention to manage the addictive smartphone behavior of this vulnerable group.

Smartphone behavior includes the daily duration and frequency of smartphone use and types of functions used. The findings among studies about the pattern of using smartphone in adolescents vary with the countries of origin and target groups [9]. Social networking is one of the popular smartphone functions being used most by adolescents and adults [11–15]. Some evidence shows that adolescents using smartphones for social networking are more likely to become smartphone addicts [11, 15]. Watching television content online with a smartphone is popular among younger children in Europe and the United States [14, 16]. Other activities such as seeking information, completing homework and killing time are common reasons youngsters stay online [1, 8, 16, 17]. Department of Health (2018) conducted a focus group interview to explore the views of using the internet and electronic screen products among school students in Hong Kong [1]. It showed that playing games was a major activity to relieve students’ stress created by heavy homework. Van Deursen et al. (2015) reported that both process- and social-related gratifications were associated with addictive smartphone behaviors in adults [15].

Uncontrollable use of smartphone is problematic and will lead to addiction to the smartphone use in the worst cases. Smartphone addiction is being perceived as a unique type of behavioral addiction distinguished from internet addiction [18]. The smartphone is a portable electronic device which has the functions of a telephone as well as the capabilities of a computer. It can be used for communication; for internet-based activities; and for personal purposes through the many “apps” available [11, 19]. Symptoms of smartphone addiction are (1) smartphone is his companion, on which he/she relies to can produce pleasure and relieve stress; (2) a person is unable to control his use of the smartphone, particularly for engaging with social media, etc.; and (3) the uncontrolled use has negative effects on his/her financial, physical, psychological and social aspects [20, 21]. Park et al. (2018) reported that about 11 % of South Korean adolescents were at high risk of smartphone addiction which required further assessment and intervention [22]. Haug et al. (2015) found that 17 % of adolescents and young adults in Switzerland were addicted to smartphone [11]. In Hong Kong, 19–27 % of 11- to 18-year-old adolescents were found to be either at risk of internet addiction or were confirmed internet addicts (Shek et al., 2008; Shek & Yu, 2012) [23, 24]. Some studies showed that gender, age, family characteristics are significantly associated with smartphone addiction [15, 22, 25].

A growing body of evidence demonstrated that such smartphone addiction is associated with numerous problems in physical, psychological and social health of children and adolescents. Excessive smartphone use is found to be associated with poor sleep quality, cardiovascular risk factors, attention-deficit hyperactivity, and depression and anxiety symptoms [26–28]. It is also shown that smartphone addiction is associated with behavioral and sexual issues commonly identified in adolescents such as antisocial behaviors, smoking, alcohol consumption, more sexually active [27, 29, 30]. The significant linkage of smartphone addiction with a wide range of physio-psychosocial problems among adolescents urges nurses especially those working school and other youth health care settings to look for effective preventive measures to tackle the uncontrollable smartphone use among adolescents prior to the occurrences of negative heath consequences.

### Resilience as a potential protective factor for smartphone addiction

Previous observational studies showed that psychological traits such as resilience may better predict smartphone addition than the pattern of smartphone behavior [31, 32]. Resilience is one’s ability to maintain psychological wellbeing and adapt to stress or difficult situations successfully [33, 34]. Factors affecting resilience development include intrinsic characteristics of an individual such as strong emotion regulation, high coping self-efficacy; child’s relationships with parents and social factors such as quality of schools and neighborhoods [35]. Low resilience has been found to be associated with internet/smartphone addiction. Strong resilience was found to be a key factor in protecting adolescents from experiencing online risks. It can also neutralize the negative psychological effects associated with internet addiction and online risk exposure [36]. 9–10 year-old youngsters with low resilience had a higher risk of becoming internet addicts [37–39]. Kim et al. (2014) examined the association of depression, impulsiveness and resilience on smartphone addiction in university students in South Korea. It reported that the addiction group spent more time using smartphones on weekdays than the non-addiction group. The addiction group had a significantly lower resilience score measured by Conner-Davidson Resilience Scale than the non-addiction group [27]. Jung and Kim (2015) found that adolescents with high addiction risk had a significantly lower ego resilience than those with potential or no risk [40]. More recent studies showed that resilience have a mediating effect on smartphone dependency and depression and aggressiveness among adolescents though the mediating effects may be different between sexes in internet addiction [32, 41–43]. The current evidence suggests that resilience can play a role in modulating addictive behavior of smartphone users. Increasing one’s resilience may help to prevent the addiction. It implicates nurses to look for evidence-based intervention to strengthen resilience to minimize health issues associated with smartphone addiction.

### Mindfulness-Based Cognitive Therapy (MBCT)

Little research has been conducted so far regarding effective management of smartphone addiction [44]. It is proposed that a person’s subconscious addictive behavior may be corrected by [45, 46]. Mindfulness is a kind of meditation originating from Buddhist practice. It aims to engage an individual in a full, direct, active and continuous awareness of experienced phenomena [46]. Available evidence supports that practicing mindfulness during childhood and adolescence may facilitate healthy psychosocial development such as strengthening executive function, increasing affective self-regulation, increasing the ability to moderate strong emotional states, and improving interpersonal relationships [47–50]. Studies investigating the role of mindfulness in managing behavioral addiction have primarily focused on problem gambling and work addiction, and they have rarely targeted children and/or adolescents [45]. The effect of mindfulness on smartphone addiction is yet to be explored [51]. Nonetheless, strong emotion regulation is one of prominent psychosocial characteristics of resilience [35]. Enhancing adolescents’ emotion regulation through mindfulness-related activities may possibly help to increase their resilience and so to modulate the smartphone behavior. Lee et al. (2008) developed a mindfulness-based cognitive therapy for 8- to 12-year-old children (MBCT-C) based on the adult MBCT developed by Segal et al. (2002) [52, 53]. It was intended to increase social-emotional resilience through strengthening mindful attention of children with anxiety problems [54]. A pilot study conducted by Lee et al. (2008) showed that the MBCT-C was feasible with children and that it could relieve their attention and anxiety problems [52]. Semple et al. (2010) conducted a randomized controlled trial to evaluate MBCT-C on 25 children aged 9 to 12 years [54]. Consistent trends of reduced anxiety and depressive symptoms were observed for the participants completing a full course of the therapy. In addition, 61 % of the parents reported that fewer conduct or anger management problems were observed on their children after participating in the programme. Although the small sample size may compromise the validity of these findings, it suggests the potential of MCBT-C to manage children with emotional and behavioral problems. As such, the research team will apply this therapy with adolescents and evaluate its effect on their resilience, smartphone behavior and smartphone addictive symptoms. This research protocol presents the design of this study and discuss the potential nursing implication of the study findings.

## Methods/Design

### Aim/Objectives

The aim of this study is to examine, through a cluster randomized controlled trial, the effects of a group mindfulness-based cognitive programme on smartphone behavior, resilience and symptoms of smartphone addiction in adolescents.

The primary objective is to investigate the group by the time interaction effects of the group mindfulness-based cognitive programme on resilience, smartphone behavior and the symptoms of smartphone addiction in adolescents.

There are two secondary objectives: (1) To identify adolescents with smartphone addictive symptoms using the Smartphone Addiction Scale-Short Version; (2) To investigate the association of socio-demographic factors, smartphone behavior and resilience with smartphone addiction symptoms in adolescents.

### Hypotheses

The three primary hypotheses of this study are: (1) the smartphone behavior of adolescents can be improved by participating in a group mindfulness-based cognitive programme; (2) the smartphone addictive symptoms of adolescents can be reduced by participating in a group mindfulness-based cognitive programme; (3) the resilience of adolescents can be increased by participating in a group mindfulness-based cognitive programme. The secondary hypothesis is that socio-demographic characteristics, resilience and smartphone behavior are associated with the symptoms of smartphone addiction in adolescents.

### Study design and setting

**Fig. 1:**
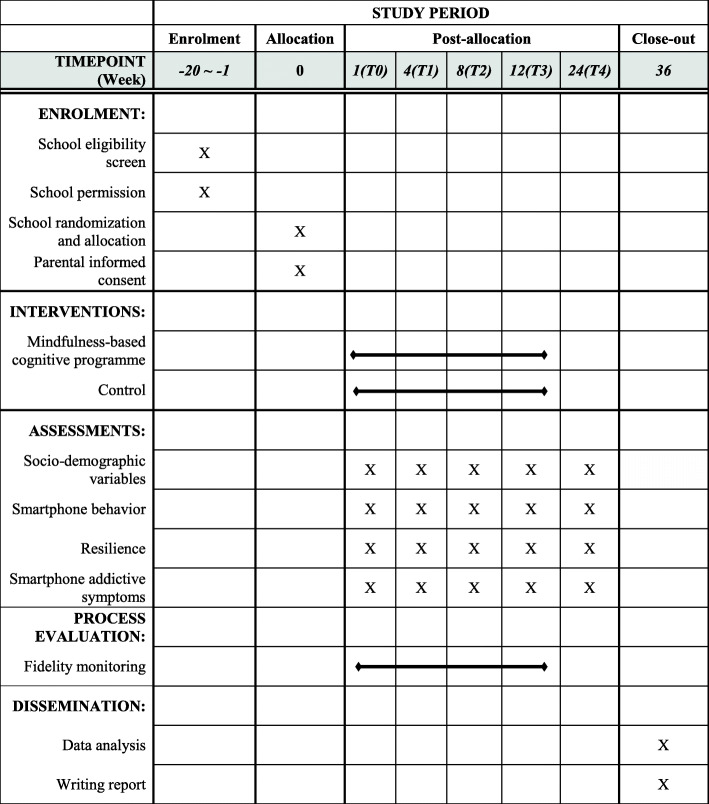
Standard Protocol Items: Recommendations for Interventional Trials (SPIRIT) Figure

This study will be an open-label, parallel-group, cluster randomized controlled trial with repeated measurement analysis. It will pragmatically evaluate the effects of the mindfulness-based cognitive programme (MBCP) on improving adolescents’ resilience, changing their smartphone behavior and reducing smartphone addictive symptoms as compared with the control group at five measurement points (i.e. baseline (T0), one month (T1), two months (T2), three months (T3) after the programme, three months’ follow up (T4)). The study will be conducted in four primary schools in Hong Kong. The primary schools will be the unit of allocation, and individual participants will be the units of analysis. The intervention group will receive the mindfulness-based programme, and the control group will receive existing counselling services for students with emotional/behavioral problems. (i.e. treatment as usual). Data collection will be started in September 2020. Key aspects of the study design can be found in Fig. 1. We designed and reported this study protocol according to the SPIRIT2013 statement.

### School and participant recruitment

Primary school will be the cluster for randomization. Four primary schools in Hong Kong will be recruited based on their eligibility and willingness to participate in the study. The inclusion criteria for school selection are: (1) it is either a local government-subsidized or a private primary school; and (2) there are currently no interventions adopted by the school to manage students’ smartphone behavior. The exclusion criteria are (1) it is an international school; and (2) there are currently interventions being implemented by the school to manage students’ smartphone behavior.

A convenience sample of 240 students will then be recruited from the selected schools (i.e., 60 students from each school) based on their eligibility and consent to participate. The inclusion criteria are: (1) adolescents are aged 10–11 years; (2) adolescents are enrolling in class level 5; (3) adolescents possess a smartphone or can access a smartphone; (4) adolescents can speak, understand and write Cantonese; (5) adolescents are cognitively capable of following simple instructions. Adolescents have intellectual/learning disabilities will be excluded from the selection.

### Sample size calculation

As it is the first study investigating the effect of the MBCP on smartphone behavior in adolescents, the project team can only decide the predicted intracluster correlation with reference to other studies investigating adolescent behavioral problems. The predicted intracluster correlation of 0.01 is adopted based on studies about smoking prevention and alcohol use in adolescents [55, 56]. 20 % potential dropout/noncompliance rate is considered reasonable. Assuming a Type 1 error of 0.05 and Type II error of 0.2, a total of 240 participants (i.e. 120 in each group, 60 in each school) is required to achieve a medium effect (Cohen’s *d*) of 0.5 [57] for comparing the treatment effect between the two treatment groups and among the measurement points within groups for the three outcomes to be measured – resilience, smartphone behavior and symptoms of smartphone addiction.

### Randomization and blinding

The four participating schools will be cluster-randomized at a ratio of 1:1 into the intervention and control groups by coin toss by the research assistant A (RA-A) who will not be involved in participant recruitment and data collection. All students recruited under the same participating school were exposed to the same intervention. The principal investigator, who will be responsible for recruiting the schools and the students, will code the participating schools with a number and a letter to represent the district and the school, respectively. RA-A will only know the four codes but not the names of the schools. The school will be assigned to the intervention group if the landed side is heads; the school will be assigned to the control group if the landed side is tails. The allocation result will be kept by the RA-A in a sealed opaque envelope until participant recruitment. The school teachers, parents and students will not be informed of the research hypotheses. RA-A will feed data into the computer in separate datasheets so that the co-investigator responsible for data analysis can analyze data without having access to information about the allocation. Hence, it is a single-blind design.

### Outcome measures

#### Demographics

Demographic variables will be gender, age, parents’ educational attainment, parents’ employment status, family structure, parenting skills. Age will be measured in continuous scale; the other variables will be measured in nominal/ordinal scales.

#### Smartphone behavior

Smartphone behavior will be operationalized in terms of duration of daily smartphone use and types of smartphone functions used. Duration of daily smartphone use will be assessed by one closed-ended question - ‘On average, how long do you use your smartphone on a typical day?’. The respondents will respond to the question by choosing among the six categories - ‘less than 10 minutes’, ’11–60 minutes’, ‘1–2 hours’, ‘3–4 hours’, ‘5–6 hours’, or ‘more than 6 hours’. For types of smartphone functions used, the respondents will indicate the frequency of using a specific smartphone function such as social networking, phone calls, gaming, etc. in a 5-point Likert scale (0 = never, 1 = rarely, 2 = sometimes, 3 = usually, 4 = always).

#### Resilience

Resilience will be measured by the Chinese Version of the Connor-Davidson Resilience Scale – 25 [58, 59]. It consists of 25 items reflecting five factors of resilience: (1) tenacity, (2) control, (3) spiritual influence, (4) positive acceptance of changes and secure relationship, and (5) tolerance of negative affect and strengthening effects of stress [58]. Each item consists of statements such as ‘able to adapt to change’, ‘close and secure relationships’, ‘sometimes fate or God can help’, ‘can deal with whatever comes’, etc. The respondents will be asked to rate their agreement with each statement based on their experiences in the previous month. If a particular situation has not arisen in that time, the respondents will be instructed to respond according to how they think they would have reacted. The respondents will be directed to indicate their response on each item with a 5-point Likert scale, 0 is ‘not true at all’ and 4 is ‘true all the time’. The total resilience score ranges from 0 to 100, with higher scores indicating greater resilience. The scale has been applied to various target populations including children 10 years old and above. A Flesch-Kincaid score of 5 indicates that the scale is expected to be understood by those with a fifth-grade level education that is 10 years of age [60]. Thus, the scale is suitable for the target group in this study. Regarding the validity and reliability of the Chinese version, the Cronbach’s alpha coefficient was 0.89. The resilience score had positive correlation with social support (*r* = 0.44) and negative correlations with depression (r=-0.38) and anxiety (r=-0.25) [59]. To ensure the scale is appropriate and comprehensible to class level 5 Hong Kong primary school students, a panel of primary school teachers will be invited to review the scale and make minor modifications in the wordings. Face validity, internal consistency and test-retest reliability will be conducted with 20 adolescents before the actual data collection.

#### Smartphone addictive symptoms

Smartphone additive symptoms will be assessed using the Smartphone Addiction Scale-Short Version. This 10-item self-administered questionnaire was developed by Kwon et al., 2013 based on the original validated Smartphone Addiction Scale [18]. The scale addresses five domains of smartphone addiction: (1) daily-life disturbance, (2) withdrawal, (3) cyberspace-oriented relationship, (4) overuse and (5) tolerance. The respondents will rate their agreement with each item on a 6-point Likert scale, from 1 representing ‘strongly disagree’ to 6, representing ‘strongly agree’. The total addiction score possible ranges from 10 to 60. The higher the score, the more severe the addictive symptoms. The internal consistency (Cronbach’s alpha) was 0.911. Analysis of receiver operating characteristics demonstrated that for boys, the sensitivity was 0.867 and the specificity was 0.893. For girls, the sensitivity and the specificity were 0.875 and 0.886 respectively [18]. A Chinese-version of the scale will be translated based on the English version by backward translation, i.e. a native Chinese and English-speaking teacher. Face validity, internal consistency and test-retest reliability will be conducted by inviting 20 adolescents to fill out the questionnaires before actual data collection, to ensure the translated version is valid and reliable for this study.

### Intervention

The participants allocated to the intervention group will receive a 12-week group mindfulness-based cognitive programme (MBCP) adapted from the MCBT-C developed by Lee et al. (2008) [52]. It consists of two components: weekly 90-minute supervised practice, and daily 15 min home practice. Supervised practice will be conducted in a group of 20 at a quiet room of the primary school. In the first supervised practice session, the trainer will inform the participants the rules of the practice sessions, for example, ‘we can remember not to talk when another person is talking.’, ‘we agree not to talk during mindful awareness practices so as not to disturb others.’, etc.

 Participants will be directed to perform a short breath meditation at the beginning and at the end of each session. To engage the young participants in the session, the trainer, who has years of experience in providing mindfulness and cognitive therapy to children, will use a variety of short sensory-based movement activities that are practiced repeatedly in 3- to 5-minute blocks. These simple sensory exercises can enhance their mindful awareness by helping them to experience internal and external environments non-judgmentally through various senses, i.e. sight, sound, touch, taste, smell and kinesthetic. Examples of these activities are sensory-based practices, seated breath meditations, mindful movement activities, body scans, visualization practices, and drawing and writing [51]. If there are participants with food allergies, the trainer will not select the allergic foods for the eating/smelling/touching activities. The parents will be informed the food that will be used for training in the week before each session in writing.

Aside from the supervised practice, the participants will be introduced to three to four home-practice activities during each supervised session. They will be encouraged to perform these activities for about 15 min a day, six days a week. Each participant will have his/her own logbook labeled with his/her code to record the home practices. The trainer will review the practice record every week. Those who have not performed the home-practice activities will be reminded by the trainer to do them in the coming week to increase the compliance. Details of the mindfulness-based cognitive programme can be found in Table 1.

**Table 1:** Mindfulness-based Cognitive Programme. (modified from Semple & Lee, 2011)[61]

Week	Supervised Practices		Home Practices (15 min/day x 6 days a week)
	Session (90 min)	Mindfulness-based cognitive technique	
1	Session 1	- Foster a secure, cohesive group environment devoted to the cultivation of mindfulness - Inform group norms and privacy of participants - Invite children to pair off and introduce themselves - Hand the half-full cup to the next child and not to spill any water - Draw on the front cover of the notebook ‘Mindfulness in Everyday Life’ - Cultivate mindfulness with breathing: Practice mindful breathing - Discussion: the differences between the experiences of mindful breathing and usual breathing	- Mindful breathing lying down - Mindful breathing sitting up - Living with awareness
2	Session 2	- Review home practices from previous session - Practice mindful breathing - Discussion: what is the purpose of practicing mindfulness in life? - Introduce mindful awareness of breath, body sensations and movements: eating, walk around the school hall	- Living with awareness - Mindful breathing - Mindful eating
3	Session 3	- Review home practices from previous session - Practice mindful breathing - Describe thoughts and feelings after hearing a short scene description - Listen the sound of bell ringing and the silence followed - Discussion: what was it like to observe the inside of the body with mindful attention? what did you notice about silence that we might not normally notice?	- Mindful breathing - Mindfulness of the body - Pleasant events
4	Session 4	- Review home practices from previous session - Practice mindful breathing - Enhance awareness to thoughts and feelings associated with food by practicing mindful eating - Understand that thoughts, feelings and body sensations are subjective experience: compare and discuss the differences between the mindful eating and the usual eating experiences - Cultivate mindfulness in body movement: practice several yoga postures	- Three-minute breathing space - Mindful yoga movements - Tasting fruits
5	Session 5	- Review home practices from previous session - Practice mindful breathing - Introduce mindful hearing: listen to music - Discussion: what made us each have different responses to the same piece of music? - Practice awareness to body sensation: pay attention to sensations in the body	- Three-minute breathing space - Mindfulness of the body - Mindful listening
6	Session 6	- Review home practices from previous session - Practice mindful breathing - Practice using sounds to express emotions: Be a conductor to guide the group to create music - Discussion: how is it that we don’t have the same thoughts and feelings when we hear the same thing? - Cultivate mindfulness in body movement: practice several yoga postures	- Three-minute breathing space - Mindful yoga movements - Unpleasant sounds
7	Session 7	- Review home practices from previous session - Practice mindful breathing - Learn to differentiate between judging and noting: practice mindful seeing - Practice mindful body movement: perform several yoga postures - Visualization with clarity: Draw a picture of the phone the children use most often - Discussion: how is seeing from your ‘Mind’s Eyes’ different from seeing with your real eyes?	- Three-minute breathing space - Seeing the little detail - Stressful events
8	Session 8	- Review home practices from previous session - Practice mindful breathing - Learn to shift attention with purpose and intention: practice mindful seeing and discussion - Differentiate descriptive observations with feelings, thoughts and body sensations: practice mindful stretching	- Three-minute breathing space - Choosing to be aware - Seeing five new things
9	Session 9	- Review home practices from previous session - Practice mindful breathing - Deepen awareness of judgement and how judgment influence one’s perceptions of the things we touch: practice mindful touch - Practice awareness to body sensation: pay attention to all sorts of sensations in the body	- Three-minute breathing space - Mindfulness of the body - Mindful touching
10	Session 10	- Review home practices from previous session - Practice mindful breathing - Practice mindful smelling: Smell the scents inside a container and describe it with one adjective - Discussion: reflection on how judgments and emotional reactivity change the nature of experience - Practice mindful body movement: perform several yoga postures	- Three-minute breathing space - Mindful yoga movements - Mindful smelling
11	Session 11	- Review home practices from previous session - Practice mindful breathing and mindful eating - Review of concepts and skills learned in previous sessions - Consolidate insights gained over the past weeks - Understand that beliefs and expectations are often inaccurate: Listen to a four sentence-story and write down what the children think about the story - Understand how emotions affect an interpretation of an event: Listen to a story, see and feel it by imaging themselves as part of it - Draw on the back cover of the notebook ‘Mindful in Everyday Life’	- Three-minute breathing space - Letter to my self
12	Session 12	- Review home practices from previous session - Practice mindful breathing - Discussion: personal experiences in the past 12 weeks; ways to continue to cultivate mindful awareness in daily lives.	------

Over the data collection period, the controls will not exposed to any interventions except counselling services that normally provided to students in need by social workers at the school due to emotional/behavioral issues.

### Safety monitoring

During the training session, another research assistant (RA-B), who is a qualified registered nurse, will closely monitor whether the participating students have allergic symptoms such as itchiness, skin rash, etc. If a student has these symptoms, the trainer will inform the responsible teacher immediately. The parents will also be contacted to explain the allergic reaction and advise them to consult their family doctor for detailed assessment. In case the student manifests severe allergic reactions, the trainer and the research assistant will follow the guidelines and emergency care plan on food allergy and anaphylaxis of the schools to call emergency service and provide basic life support to the student immediately.

### Data collection

**Fig. 2:**
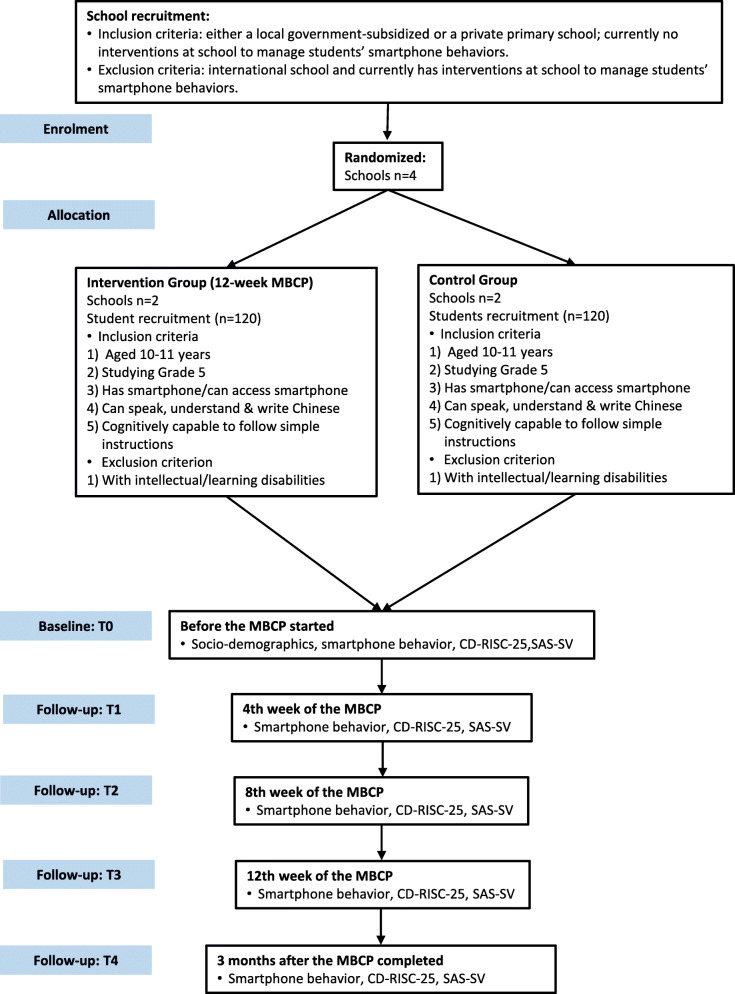
Trial Flow Chart

The information sheets, the consent forms, the questionnaires and envelopes will be distributed to the parents of the class level 5 students in the participating primary schools via class teachers. The parents will be required to indicate the food(s) their child is allergic to, if any, on the consent form. The parents who are willing to allow their children to participate in the study will return signed consent forms and completed questionnaires in sealed envelopes to the school before starting the intervention (T0). The trainer will record the attendance of each participant in every session throughout the 12-week intervention. To follow up on the effect of the intervention on the participants, post-intervention assessments will be conducted on all outcomes in week 4, week 8, by the end (week 12) of the programme and 3 months follow-up (T1-T4). It will take about 20 min to complete the questionnaire. RA-B will be responsible for collecting all the questionnaires. The school teachers will not be involved in the data collection. Figure 2 shows the trial flow.

### Validity and reliability/rigour

To ensure intervention fidelity and adherence, structured observations of a randomly selected session per week will be performed by RA-B. The project team will first develop quality scales to assess the fidelity quantitatively. The quality scales will be session-specific. For the home practice, each participant will have a logbook to record their compliance to the recommended home-practice activities. In addition to the reminder to the participants regarding the home practices by the end of every supervised session, a circular will also be sent to their parents to solicit their help to monitor their children for the home practices every week to increase the treatment adherence.

As both participants in the intervention and control groups are allowed to receive counselling services provided by the school social workers if necessary to handle some emotional/behavioral issues, a record of whom have received the service and how often will be kept to facilitate an interpretation of the study findings.

### Data analysis

IBM SPSS version 23 will be used for statistical analysis. Mean and standard deviation will be reported for continuous variables, i.e. age, types of smartphone functions used and total resilience score. Frequency and percentage will be reported for nominal and ordinal variables. Cronbach’s alpha and intra-class correlation coefficients will be calculated to determine the validities and reliabilities of the two scales: the translated Smartphone Addiction Scale and the Chinese Version of Conner-Davidson Resilience Scale – 25. Independent t-test/Mann-Whitney U test will be used to compare the socio-demographic variables between intervention and control groups. Generalized Estimating Equation model will be used to verify all the hypotheses. The socio-demographic variables will be treated as co-variates to adjust the group means. Results will be reported with 95 % confidence interval. Intention-to-treat analyses will be applied to the independent and dependent variables. Missing data will be handled by replacing the missing values with the item-specific mean. Tests performed will be two-tailed with alpha value set at 0.05. Cohen’s *d*, calculated by taking the difference of the adjusted means between two comparison groups and dividing it by the pooled standard deviation, will be used to estimate the effect size of the treatment.

## Discussion

The expected outcome of this study is that the mindfulness-based cognitive programme will lead to improvements in resilience and reduction of smartphone addiction risk among adolescents. The MBCP enables the adolescents to grasp a new skill to manage their emotion by attending to the present and modify their cognitive thoughts toward stress and difficulties encountered in life. Throughout the training process, the adolescents will learn that they can manage their emotion through daily mindfulness practice. The programme has a lifelong benefit to adolescents as it enables them to manage their stress and conflicting thoughts which linger in adolescents with a simple and economical approach.

Problematic smartphone use, when not managed timely and adequately, may progress to smartphone addiction and other associated negative health outcomes. Our intervention can be served as a preventive intervention to be applied regularly in schools and other adolescent health centers to strengthen adolescents’ resilience in order to boost up their resistance against addictive smartphone use. Nurses, being one of the major frontline healthcare providers in the community to monitor people’s health and provide health promoting intervention, could integrate this noninvasive and simple-to-administer intervention into existing adolescent wellness programmes to not merely manage smartphone use issue but also to promote the overall wellbeing of the adolescent clients.

It is hoped that findings from the study could lead to larger, multisite trials to provide more robust evidence. In addition, similar evidence-based approaches can be developed in the future for other age groups who are vulnerable to smartphone addiction to strengthen the protection against the negative effects of smartphone use.

There are several limitations of this protocol. This study targets early adolescents; the generalizability of the study findings to middle- and late-adolescents is unknown. As two treatment groups will receive treatments in totally different formats, ascertainment bias might be introduced to the results although the study hypotheses will not be made known to the schools, the parents and the students. Only four primary schools will be recruited; the similarity in characteristics exhibited by participants from the same school may bias the results. To assess the impact of potential clustering of the participants from the same school, Generalized Estimating Equation model will be adopted to control the cluster effect of school.

## Data Availability

Not applicable.

## Abbreviations

MBCT: Mindfulness-based cognitive therapy.
MBCT-C: Mindfulness-based cognitive therapy for children.
MBCP: Mindfulness-based cognitive programme.
RA-A: the first research assistant.
RA-B: the second research assistant.
CD-RISC-25: Connor-Davidson Resilience Scale − 25.
SAS-SV: Smartphone Addiction Scale–Short Version.
